# Increasing community health worker productivity and effectiveness: a review of the influence of the work environment

**DOI:** 10.1186/1478-4491-10-38

**Published:** 2012-09-27

**Authors:** Wanda Jaskiewicz, Kate Tulenko

**Affiliations:** 1 Capacity Plus, IntraHealth International Inc, 1776 I Street NW, Suite 650, Washington DC, USA, 20006

## Abstract

**Background:**

Community health workers (CHWs) are increasingly recognized as a critical link in improving access to services and achieving the health-related Millennium Development Goals. Given the financial and human resources constraints in developing countries, CHWs are expected to do more without necessarily receiving the needed support to do their jobs well. How much can be expected of CHWs before work overload and reduced organizational support negatively affect their productivity, the quality of services, and in turn the effectiveness of the community-based programmes that rely on them? This article presents policy-makers and programme managers with key considerations for a model to improve the work environment as an important approach to increase CHW productivity and, ultimately, the effectiveness of community-based strategies.

**Methods:**

A desk review of selective published and unpublished articles and reports on CHW programs in developing countries was conducted to analyse and organize findings on the elements that influence CHW productivity. The search was not exhaustive but rather was meant to gather information on general themes that run through the various documents to generate perspectives on the issue and provide evidence on which to formulate ideas. After an initial search for key terminology related to CHW productivity, a snowball technique was used where a reference in one article led to the discovery of additional documents and reports.

**Results:**

CHW productivity is determined in large part by the conditions under which they work. Attention to the provision of an enabling work environment for CHWs is essential for achieving high levels of productivity. We present a model in which the work environment encompasses four essential elements—workload, supportive supervision, supplies and equipment, and respect from the community and the health system—that affect the productivity of CHWs. We propose that when CHWs have a manageable workload in terms of a realistic number of tasks and clients, an organized manner of carrying out these tasks, a reasonable geographic distance to cover, the needed supplies and equipment, a supportive supervisor, and respect and acceptance from the community and the health system, they can function more productively and contribute to an effective community-based strategy.

**Conclusions:**

As more countries look to scale up CHW programmes or shift additional tasks to CHWs, it is critical to pay attention to the elements that affect CHW productivity during programme design as well as implementation. An enabling work environment is crucial to maximize CHW productivity. Policy-makers, programme managers, and other stakeholders need to carefully consider how the productivity elements related to the work environment are defined and incorporated in the overall CHW strategy. Establishing a balance among the four elements that constitute a CHW’s work environment will help make great strides in improving the effectiveness and quality of the services provided by CHWs.

## Background

Community health workers (CHWs) are increasingly recognized as an integral part of the health workforce needed to achieve the health-related Millennium Development Goals [1]. CHWs are a critical link in increasing communities’ access to services, especially for those people living in rural and underserved areas. Accordingly, the Global Health Workforce Alliance commissioned a systematic review to gather the latest evidence on wide-scale use of CHWs. This was followed by a consultation meeting in April 2010 to review recommendations and reach agreement on key messages for country-level integration of CHWs into the health workforce.

CHWs are frequently called upon to address a number of essential service delivery needs, including maternal and child health, family planning, human immunodeficiency virus (HIV) and acquired immunodeficiency syndrome (AIDS), malaria, and environmental health. As task-shifting becomes more widely implemented, CHWs have more tasks piled on to their list of job responsibilities. A multi-country study noted “an evolution over time, whereby CHWs typically take on additional responsibilities and skills, which are learned on-site” [2]. Given the serious financial and human resources constraints in developing countries, CHWs are expected to do more although they may not always receive the necessary support to do their jobs well, such as supportive supervision, and supplies and equipment. There is still no resolution to the long-standing debate on the question of how many functions an individual CHW can realistically and effectively perform within his or her approved scope of practice [3-6]. Naturally, there is a limit to productivity such that when the workload is pushed beyond a certain level, CHW performance will suffer. There is a question as to how much can be expected from CHWs before the work overload and reduced organizational support negatively affect their productivity and the quality of their services, and in turn the effectiveness of the community-based programmes that rely on them.

Relatively little attention has been given to the issue of CHW productivity, although the “benefits of addressing productivity include greater efficiency, reduced workload, increased job satisfaction, and higher quality of care” [7]. As more countries and nongovernmental organizations incorporate CHW strategies into their health programmes, the need grows for guidance on how to maximize investments in CHW programmes in terms of productivity and its effect on quality. This article presents policy-makers and programme managers with key considerations for a model to improve the work environment as an important approach to increase CHW productivity, and ultimately the overall effectiveness of community-based strategies.

For the purposes of this article a CHW is defined as a “health worker who performs a set of essential health services and who receives standardized training outside the formal nursing or medical curricula and has a defined role within the community and the larger health system” [8].

## Methods

A desk review of selective published and unpublished articles and reports on CHW programmes in developing countries was conducted by CapacityPlus, the United States Agency for International Development's (USAID) global human resource for health project to analyse and organize findings on the elements that influence CHW productivity. The search was not an exhaustive attempt to uncover all writings on the topic but was meant to gather information on general themes that run through various documents to generate perspectives on the issue and provide evidence on which to formulate ideas. After an initial search for key terminology related to productivity, a snowball technique was used where a reference in one article led to the discovery of additional documents and reports to be reviewed.

This paper has a narrow focus on the elements of CHW productivity and does not explore links between productivity and the resulting performance of CHWs. A discussion on associations between CHW productivity and performance and a programme’s impact on the health of the populations served by CHWs is beyond the purview of this paper.

## Results and discussion

Little has been published that specifically addresses the productivity of CHWs; most studies concentrate on the performance of CHWs or the overall CHW programme or strategy. However, we can extrapolate from the theories and perspectives describing the productivity of facility-based health workers. From an economic standpoint, productivity is often defined as the ratio of outputs to inputs [9] or the ratio of what is produced to what is required to produce it [10]. These economic definitions of productivity have been translated for the health sector as the services provided by a health worker over a given period of time [11]. This paper borrows from the economic perspective to discuss some of the key requirements or inputs needed for CHWs to be productive in providing essential health services (outputs) to the communities they serve.

For a CHW (or any health worker) to be productive, a number of broad-based and interrelated inputs are required. These include:

capacity (knowledge, skills, and attitudes)

motivation

organizational support or the “opportunity to do the job well” (resources, physical and social environment, working conditions) [12-14].

**Figure 1:**
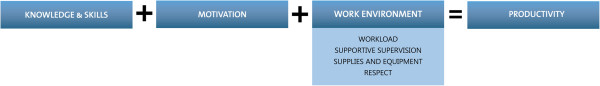
Work environment as a key determinant of community health worker productivity.

While the capacity and motivation (both extrinsic and intrinsic) to do the work are also essential determinants of a CHW’s productivity, these are areas that have already been extensively covered in published literature. This paper will focus only on describing the third category of organizational support, with specific attention to the provision of an enabling work environment that is conducive to high levels of productivity. An enabling work environment is a general term to describe the inputs or considerations needed from the institution engaging CHWs and represents the conditions under which CHWs perform their duties. The work environment or working conditions encompass four key elements—workload, supportive supervision, supplies and equipment, and respect [15]—that affect the productivity of CHWs. This model of productivity is illustrated in Figure 1.

“Working conditions, part of the broader human resources management system, are important in terms of creating the conditions for effective and efficient work, boosting morale, and reducing turnover and attrition” [16,17]. Lack of attention to working conditions and human resources management is a key factor in the foundering of CHW programmes [17]. One key message from the Global Health Workforce Alliance consultative meeting on CHWs emphasizes the need to “ensure a positive practice environment, including regular and continuous supportive supervision, health and safety issues, CHW’s information and communication needs, a clean environment, a manageable workload, and the availability of drugs, supplies, and equipment” [18].

### Workload

**Figure 2:**
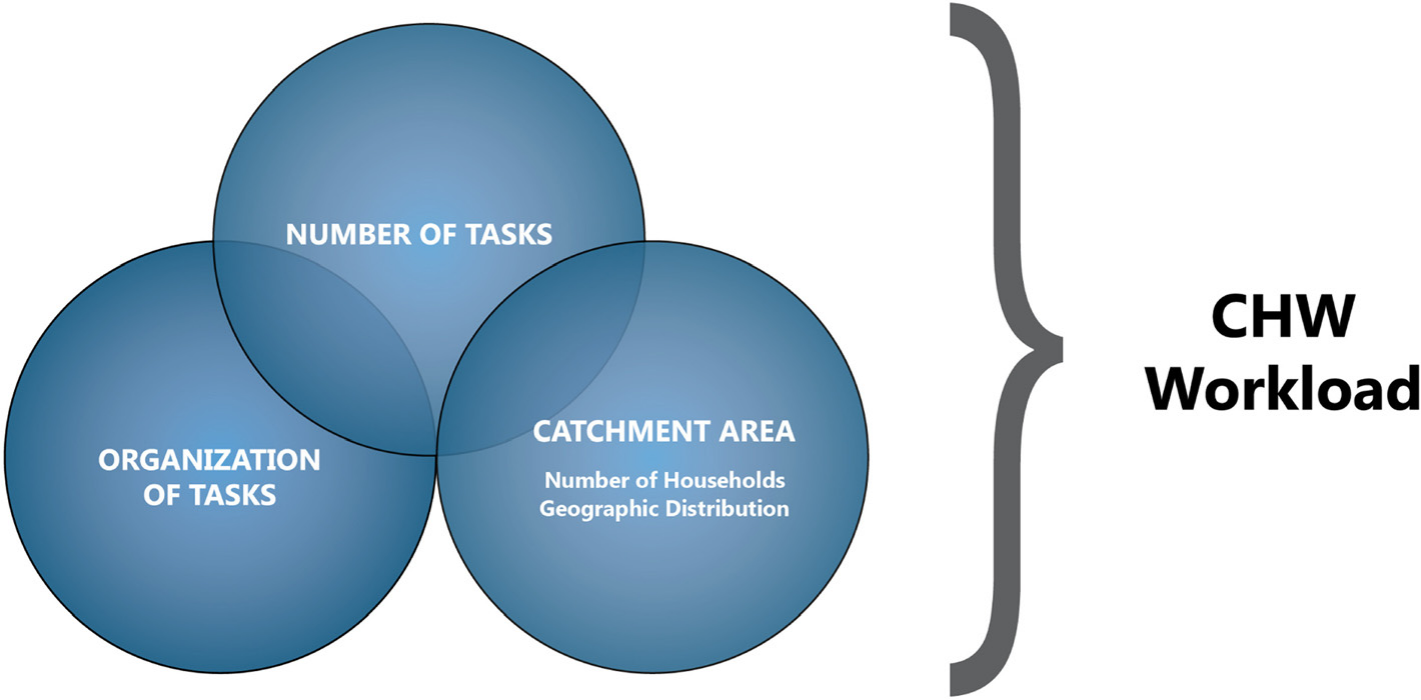
Components of community health worker workload.

As described above, workload plays a defining role in the level of productivity and quality that can be expected of CHWs. Workload is a multifactorial concept that can be described by the interplay of the number and organization of tasks and the catchment area. The catchment area can be further divided into two equally important aspects: the number of households to be served and their geographic distribution (see Figure 2). To ensure a realistic workload, all of the subcomponents must be considered in turn.

#### Number of tasks

There is no known ideal or maximum number or mix of CHW job tasks that will ensure the highest level of CHW productivity. However, much has been reported both anecdotally and empirically regarding the consequences that too many responsibilities can have on CHW productivity and consequently on the quality of the services they provide. Evaluations have reported that CHWs often become “overwhelmed by a very broad range of tasks with negative effects on the overall quality of their performance” [19]. A study of the working conditions of health extension workers in the Federal Democratic Republic of Ethiopia found that most work long hours, including on Sundays [17]. A qualitative study of Lady Health Workers in the Islamic Republic of Pakistan illustrated that the addition of responsibilities not in their job descriptions, such as involvement in polio eradication campaigns, loading and unloading of medicines, and transportation of stocks, took valuable time away from their regular work [20].

Generalist CHWs, whose duties encompass a wide range of service delivery tasks, tend to have the heaviest workload in terms of the number of tasks they are asked to perform. However, this may not always be the case, especially for CHWs focusing on such specific yet comprehensive health areas as HIV and AIDS. A study of CHW contributions to HIV service delivery across five countries described more than 100 possible types of tasks divided across 12 categories of care, such as education, counselling and testing, follow-up, and psychosocial support [2]. The CHW Assessment and Improvement Matrix, developed by the USAID Health Care Improvement Project, delineates a vast number of discrete tasks approved for CHWs within maternal and child health, reproductive health, nutrition, and HIV and AIDS, based on an extensive literature review of CHW scopes of practices from many countries around the world [8].

According to a literature review on CHWs, “despite the wide range of tasks that CHWs can do, they cannot do everything—their limited educational background and training mean that they can only be expected to perform a limited number of tasks that complement the work of health professionals” [21]. When there are too many tasks to perform, CHWs may not perform them all but instead select a few that they prefer to do, ones that they do best, or those that are most feasible [19]. In particular “unpaid volunteers must have a limited set of tasks and not be expected to work more than a few hours a week; otherwise they tend to abandon their responsibilities” [22]. A study on the role of health surveillance assistants (HSA) in the Republic of Malawi showed that they do not perform all the tasks in their job description, which include a plethora of activities such as vaccination, growth monitoring, disease surveillance, health education, tuberculosis follow-up, family planning provision, treatment for common diseases, and supervision of traditional birth attendants [23]. An assessment in Pakistan showed that Lady Health Workers become stressed in their job because they have little say regarding their increasingly expanding job scope and are seldom consulted when their job description changes [24].

Success is more likely when CHWs have a clear job description that defines a limited number of tasks [25]. CHWs can “perform better with clearly defined roles and a limited series of specific tasks than if they are expected to undertake a wide range of tasks or have an ill-defined role” [26]. Clearly defined roles, standardized protocols, and job aids should ensure that CHWs practise within the limits of what they can achieve and for which they have been trained [21]. In the Sultanate of Oman, where community support group volunteers have a limited job description, their coverage was high; 80% of women surveyed reported contact with the volunteers [21,27].

Programmes must “avoid over-burdening CHWs with competing priorities and expanding interventions of various initiatives” [26] without making concessions in other aspects of their work environment. For example, it may be possible to increase the range of services provided by CHWs if other adjustments are made, such as reducing the catchment population, increasing their capacity with training, and providing stronger supportive supervision. Programmes must carefully assess and monitor the workload of CHWs and the effect on CHW motivation and productivity as more tasks are added to their list of job responsibilities [5]. Monitoring and assessments of CHW programmes should regularly include checking in with CHWs to obtain their feedback on programme inputs and suggestions for improvements. Listening to CHWs is valuable to glean their perceptive insights on how their work environment can be organized for maximum productivity and also as an intrinsic motivator.

#### Organization of tasks

Beyond the actual number of tasks assigned to a CHW, the organization of those tasks can assist in maximizing productivity. For example, if a task needs to be conducted only once or twice a year, such as providing deworming tablets, it does not have much impact on other tasks that are carried out on a more regular basis. Likewise, the manner in which CHWs are trained to carry out the various tasks can influence productivity. For example, a modified version of systematic screening, used by facility-based professional health workers, could be adapted to increase the number of services provided at a single client visit. In this way, the CHW would use a checklist, questionnaire, or other job aid to ask the client about health areas within the CHW’s scope of practice, in order to identify the client’s needs and where possible provide all the services or information within that visit or refer to the next level. Multi-country studies (with professional health workers) have shown that systematic screening can increase the number of services received per client visit by 9% to 35% [28,29]. The screening approach, if appropriately adapted for a CHW programme, could increase efficiency by decreasing the number of visits to the households, preserving clients’ time, and reducing transport and other costs.

Another strategy is to integrate services provided by CHWs to meet the broader health service needs of the community. A survey of community-based reproductive health agents in Ethiopia found that integrated service delivery appears to increase the amount of time that agents must spend with each client [30]. Where productivity is defined as time spent with a client, this integrated delivery approach would increase productivity and possibly the satisfaction of clients and the quality of services.

#### Catchment area

The amount of work that a CHW’s catchment area entails depends on the number of households each CHW is responsible for, the target group within the family (e.g. all family members, children only, women only), as well as the geographic distribution of those households. A critical question regards the optimal population size that a CHW could cover [31]. No set formula exists for the optimal number of households CHWs can feasibly serve with a minimum standard of care. There are countries, such as the Democratic Socialist Republic of Sri Lanka, where a CHW covers as few as 10 households with maternal and child health services, while in India a CHW covers about 1000 households (approximately 5000 population, usually spread over 5 to 10 villages) [32]. One of the factors of the Care Group model’s effectiveness in reducing infant and under-five mortality in Gaza province, the Republic of Mozambique, was assigning each volunteer (part of a group of 10–15 village volunteers in an extensive network of 2300 volunteers) responsibility for health promotion activities and registration of vital events for only 10 neighbouring households [33]. The population coverage and the range of services offered at the community level are vital in the design of effective CHW schemes, and it should be noted that the “smaller the population coverage, the more integrated and intensive the service offered by the CHWs” [31].

How far apart the households are, how much geographic area they cover, the type of terrain, and whether reliable transport is available all affect how well CHWs are able to meet their performance expectations. When catchment areas are too large, CHWs may have difficulty finding the time or transportation needed to visit all the assigned households [5]. As compared with facility-based providers who spend unproductive time waiting for clients [7,28], CHWs log unproductive time getting to the client or arriving at the household to find the client absent. HSAs and senior HSAs in Malawi cover wide catchment areas, on average 5–10 km for HSAs and 10–20 kmfor senior HSAs [23]. Catchment areas where families live spread out over wide distances with difficult terrain to cross or where CHWs are not provided with appropriate transport increase the time spent on the road and decrease productivity. CHWs participating in the delivery of a community-based newborn care intervention package in the People’s Republic of Bangladesh’s Sylhet District “attended less than 5% of all births because of their high workload, travel distances, and difficulty receiving timely notification of deliveries” [34].

Programmes must take care to monitor the catchment area assigned to CHWs to ensure that they can satisfactorily reach all the targeted members within the specified geographic area with a standard level of quality of care.

### Supportive supervision

To be successful, CHW programmes require regular and reliable support and supervision [3-5]. Offering CHWs supportive supervision within the structures and functions of the health team demonstrated better outcomes [2]. Another important factor contributing to the success of the Care Group model in increasing use of child health services and reducing child mortality in Gaza province, Mozambique was the supportive environment created among the volunteers within a “care group” as well as the close supervision from programme staff, which not only “enabled appropriate decision-making by volunteers but also influenced their motivation and retention resulting in a very low dropout rate among village volunteers” [33].

Yet supervision is often one of the weakest links in a CHW programme [3]. Quality of supervision matters a great deal: ineffective supervision contributes to low CHW morale and poor productivity [35]. The following are a few examples:

Supervision of CHWs in the Republic of Zambia’s Kalabo District did not have a positive impact on performance because the quality was poor and almost half the CHWs did not experience any benefit from the supervision visits [36].

An evaluation in the Federal Republic of Nigeria found that the majority of CHWs were not engaging in such critical components of the primary health care programme as home visiting due in part to inadequate or infrequent supervision [35].

In a few of the CHW programmes in the Global Health Workforce Alliance review, supervisors were formal health staff from the health services who may not properly understand the CHWs’ roles and may resent the additional task of supervision [1].

In some evaluations that have documented weak supervision in national CHW programmes, the CHWs do not even know who their supervisors are or what they can expect from them [37].

Many health professionals lack the background to provide a supportive environment for CHWs [26]. The traditional supervisory approach that most are familiar with is more of a bureaucratic exercise, often is of limited value, and relies on a “policing” function that solely penalizes workers. What is needed is a change toward a more participatory and enabling supervisory approach that helps CHWs identify their challenges and implement solutions, and even considers using alternative technologies such as mobile phones and peer-to-peer support to create a two-way flow of information and communication. The Joint Learning Initiative Paper on CHWs in Africa emphasizes the following proposals to strengthen the supervisory approach:

"“Clear strategies and procedures for supervision and the activities with which supervisors will be charged should be well defined. The skills need to be taught so that health personnel, CHWs and community health committee members know what is expected of them as supervisors. Supervision should be taught to be undertaken in a participatory manner. Top-down mechanistic supervision emphasizes the social distance between supervisor and supervisee and leads to communication breakdowns and ultimately to programme damage. The guidelines for supervision should include a list of supervisory activities. The most important element of supervision is ensuring the two-way flow of information. It is also vital that the supervisor acts as a role model so that their behaviour can be copied." [37]

A supervisory strategy that has been effectively used in some CHW programmes, such as Bangladesh Rural Advancement Committee (BRAC)’s *Shasthya Shebikas* in Bangladesh is to assign CHWs with a higher level of training (*Shasthya Kormis*) to supervise the CHWs [38]. Unlike professional health workers, these supervisors can fully relate to the expectations, pressures, and context in which CHWs perform their duties as they themselves have experienced them first-hand. An added benefit to this supervisory approach is the effect that creating a career pathway can have on CHW motivation.

### Supplies and equipment

To carry out their tasks effectively, CHWs need a regular replenishment of supplies, medicines, and equipment. Unfortunately, this is another weak link [3]. When the supply of needed materials is disrupted not only will productivity decrease but there may be other equally detrimental consequences, such as losing the respect of the community without which a CHW can rarely be productive.

In Pakistan, “poor supply caused embarrassment and made "Lady Health Workers" suspect in the eyes of the community because they were accused of selling drugs and contraceptives in the market” [20]. CHWs need the trust of the community; when this is compromised CHWs become ineffective. In Kalabo District, Zambia, one of the two most important factors behind the dysfunction of the CHW programme was the shortage of drugs [36].

The cost of travel is an important determinant of CHW effectiveness [39] and should be factored in when considering how the supplies, materials, and equipment that CHWs need will be replenished. For example, lack of transport prevented some HSAs in Malawi from covering some of the villages in their catchment areas and from obtaining drugs and other needed supplies from their respective health centres [23].

### Respect

Acceptance, support and respect from both the community and the formal health system are essential for CHWs to be effective. While respect from the community is a key criterion for initial selection of CHWs—and indeed many CHWs are nominated by their own communities [1]—in large part the organization or formal health system engaging CHWs has the responsibility for ensuring that the initial acceptance and support is maintained. The continued respect that CHWs earn from the community relies on many factors over which the institution has a high degree of influence, such as:

– The respect given to CHWs by the health system, which can be characterized by how well the CHW contribution is accepted and understood by facility-based health workers. This often is defined by the degree to which facility-based health workers respond to CHW referrals. If community members perceive that their CHWs’ referrals are not respected, they may lose trust in the CHW and not seek further services or heed future referrals [40].

– Ensuring a reliable stock of medicines and other needed supplies, as described above.

– CHW competence, as defined by the knowledge and skills acquired through training as well as monitoring and follow-up through supportive supervision. Community perception of CHW knowledge, skills, and overall ability to help them with their health needs is important for inspiring their respect and acceptance of CHW services.

### The work environment: interplay of productivity elements

CHW productivity is influenced by a complex interplay of the four elements that comprise an enabling work environment—workload, supportive supervision, supplies and equipment, and respect. Appropriate incorporation of these elements in a CHW programme provides CHWs with the working conditions conducive to doing their job more effectively.

However, there is scant empirical evidence regarding which element of the work environment is the most important, or the exact degree to which one element or a combination of elements has a larger or smaller influence on the overall work environment and, in turn, CHW productivity. On some level, ensuring the appropriate work environment can be a balancing act and is quite programme- and context-specific. For example, BRAC’s *Shasthya Shebikas* in Bangladesh have a broad set of job responsibilities to accomplish and yet are considered highly productive and effective in their work [41,42]. *Shasthya Shebikas* cover 250–300 households within a small neighbourhood in their villages, enjoy a high level of respect from the government health sector, and receive strong supervision by higher level CHWs (*Shasthya Kormis*)*,* which includes going along on household visits to assess and support performance, monthly refresher trainings to update knowledge and problem-solve, and regular opportunities to restock drugs and supplies [1,38]. It seems clear in this case that the CHWs’ workload does not impede productivity as the other enabling elements are abundant. What is unclear is which of these elements makes the most significant difference in the *Shasthya Shebika* programme or which of the elements interrelate the most and practically predict the positive productivity outcome. Is high productivity due to the supervisory support or more so to the refresher trainings that foster competence and ensure monthly refills of needed supplies and thereby continued earning of the goodwill of the community? Is productivity bolstered more by the relatively limited geographical reach of households to cover, or by the obvious esteem of the health sector and facility-based health workers that raises the CHWs acceptance and respect in the community, which then manifests in health-seeking behaviours? Or is it some combination of two or three of these factors or others? These are the types of questions that require further research to fully understand the determinants of CHW productivity.

Alternatively, in cases where organizational support may be lacking and the work environment is less conducive, one can envision how increasing the quantity of services a CHW is expected to carry out can hamper productivity and consequently the quality of service provision. Likewise, supportive supervision without provision of the needed drugs and supplies will be inadequate as CHWs will not have the tools to properly deliver their services and will lose the respect of the community. In the same manner, limiting the quantity of expected job tasks to a manageable number while demanding an overwhelming geographical and household coverage will limit a CHW’s productivity. Conversely, if a CHW has a small and focused assignment of households within a limited geographical reach or is provided transport to more quickly and easily move across larger distances to reach target households, we may likely witness an increase in productivity because the CHW is able to fulfil a greater number of service needs in the same time period.

### Recommendations

Based on our findings we propose the following recommendations for policy-makers, programme managers, and researchers to contribute to increased CHW productivity:

Carry out further research on CHW programmes recognized to be the most effective to define the degree to which each component of productivity (including knowledge and skills and motivation, which were not discussed in this paper) and the interplay between them influences CHW productivity to determine which combination of elements is the most critical for overall CHW effectiveness.

Conduct operations research to begin to answer the question of how large a workload a CHW can undertake before productivity suffers, and in particular determine the ideal number or highest limit of tasks as well as target geographical and household coverage.

Involve CHWs in the decision about whether to add new services to their portfolio and if so, which service delivery tasks would be highly demanded and most effective.

Employ the observational technique of time-use studies to understand how CHWs use their time to carry out assigned duties and what obstacles they encounter to develop interventions for increased productivity and efficiency.

Improve the supervisory system to support CHW performance and productivity, provide recognition and feedback, assist in problem-solving, and link CHWs to the formal health sector. Seek CHW feedback on what is working and what needs to be improved in their support system or work environment.

Explore the feasibility of the use of mobile technologies to improve connectedness and communications with CHWs and as a complement to supportive supervision. Mobile phones can also improve in-service training opportunities as well as enable CHWs to more efficiently order needed supplies or refer patients.

Ensure consistency in the provision of supplies, equipment, and transport fundamental to CHW tasks.

Strengthen human resources management systems to facilitate a standard level of working conditions that foster good performance within an enabling work environment.

## Conclusions

As more countries look to scale up CHW programmes or shift additional tasks to CHWs, it is critical to pay attention to the elements that affect CHW productivity in the design phase as well as throughout implementation of a programme. An enabling work environment is crucial to maximize the productivity of CHWs. Policy-makers, programme managers, and other stakeholders need to carefully consider how the productivity elements of workload (number and organization of tasks, and number and distribution of households), supportive supervision, availability of supplies and equipment, and respect are defined and incorporated in the overall CHW strategy.

Establishing a balance among the four elements that constitute a CHW’s working environment will help make great strides in improving the effectiveness and quality of the services provided by CHWs. When CHWs have a manageable workload in terms of a realistic number of assigned tasks and clients to serve, an organized manner of carrying out these tasks, a reasonable geographic distance to cover, the needed supplies and equipment, the support and guidance of an effective supervisor, and the respect and acceptance from the community and the health system, they can function more productively.

## Competing interests

The authors declare that they have no competing interests.

## Authors’ contributions

WJ analysed the collected reports and articles from the selective desk review and wrote the journal article. KT conceived of the idea of the paper and assisted in critical thinking and technical revision. Both authors read and approved the manuscript.
